# Proteus mirabilis: A rare cause of pneumonia, radiologically mimicking malignancy of the lung

**DOI:** 10.1002/ccr3.7937

**Published:** 2023-09-19

**Authors:** Saif ullah, Ramin Saadaat, Hidayatullah Hamidi, Ahmed Maseh Haidary

**Affiliations:** ^1^ Department of Internal Medicine French Medical Institute for Mothers and Children (FMIC) Kabul Afghanistan; ^2^ Department of Pathology and Clinical Laboratory French Medical Institute for Mothers and Children (FMIC) Kabul Afghanistan; ^3^ Department of Radiology French Medical Institute for Mothers and Children Kabul Afghanistan

**Keywords:** lung cancer, mimicking, pneumonia, proteus mirabilis

## Abstract

**Key clinical message:**

Lesions that are suspected for malignancy need be managed by a multidisciplinary team. Utilization of radiological as well as pathological diagnostic modalities ensures correct diagnosis and thus timely intervention.

**Abstract:**

**Introduction:**

*Proteus mirabilis* is a Gram‐negative rod. It is a highly motile bacterium that belongs to the Enterobacteriaceae. Lung infection and pneumonia caused by *p. mirabilis* is extremely rare and occurs in patients with chronic debilitation or chronic lung disease.

**Case Presentation:**

A 65‐year‐Old Woman presented with dry cough, dyspnoea on exertion, and chest pain of 4 months' duration. She received multiple medications including antibiotics but without any resolution of her symptoms. Computed Tomography scan of the chest was performed reported a tumor in the upper lobe of the left lung with multiple associated pulmonary nodules. The impression was that of metastatic lung disease with superimposed acute infection. Accordingly, the patient was reevaluated and a diagnostic bronchoscopy with multiple endobronchial biopsies and broncho‐alveolar lavage was done. The gram stain showed Gram‐Negative Bacilli and the bacteria identified *P. mirabilis*.

**Conclusion:**

Mass lesions suspected for malignancy should be managed with involvement of multiple medical disciplines, to ensure correct and timely diagnosis. This is to avoid miss‐management.

## Graphical abstract

*Proteus mirabilis* pneumonia, radiologically mimicking lung cancer.

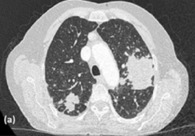

## 1 INTRODUCTION

*Proteus mirabilis*, belongs to the *Enterobacteriaceae* family of bacilli. It is a gram‐negative, facultative anaerobe and has an ability to ferment maltose but inability to ferment lactose.^1^ Considering biochemical properties, *P. mirabilis* is urease‐positive, lactose‐negative, indole negative and hydrogen sulfide producing microorganism. Urease production and robust swarming motility by the production of polysaccharide that allows it to attach to surfaces and thus to elongae, are the two hallmark features of this organism. Additionally the bacterium has fimbriae on its surface that allows for its ability to attach to surfaces.^1^

*P. mirabilis* is a highly motile bacterium, that causes swarming on the culture medium.^2^ Although *P*. *mirabilis* can be found in a wide range of settings, such as soil, water sources, and sewage. It is mostly a commensal of both human and animal gastrointestinal tracts.^3^

*P. mirabilis* rarely causes lung infection and pneumonia because it does not have high virulence, however in opportunistically and in immunosuppressed patients it can causes wound infection, peritonitis, urinary tract infections, biliary tract infections that can lead to systemic infections, and in rare situations patient can develop pneumonia.^4^ Such a presentation can occur in patients with chronic debilitation, chronic lung disease, in alcoholics, in individuals with renal failure and frail elderly individuals. Other predisposing factors for pneumonia include prolong use of antibiotics especially flouroquinolones and cephalosporins, corticosteroids and immunosuppressive agents and mechanical lung ventilation.^5^ Here we present a rare case of *p. mirabilis* induced pneumonia in a patient who presented with a lesion in the lung that was suspected to be lung cancer. Multidisciplinary management approach allowed for correct diagnosis and appropriate treatment.

## 2 CASE PRESENTATION

A 65‐Year‐Old Woman, presented to the outpatient department with chief complains of dyspnea on exertion, cough and chest pain of 4 months' duration. Dyspnea was gradual in onset that exacerbated on exertion and relieved at rest. At the time of presentation, the dyspnea it was grade 2 based on modified medical research council (MMRC) dyspnea scale. Cough was dry at the onset with occasional mucopurulent secretion mixed with fresh blood. The chest pain was gradual in onset, involving the left side, initially intermittent but later became persistent with mild to moderate severity, exacerbated on exertion and relieved by over‐the‐counter analgesics. Past history was significant for biomass exposure as she had history of exposure to wood smoke during adolescence. Similarly, she was chronic hypertensive and regularly used antihypertensive medications. Before presentation to our institution, the patient was treated with multiple courses of antibiotics, including amoxicillin‐clavulanic acid, ceftriaxone, cefepime, and ceftazidime with no improvement.

On General Physical Examination, Patient was ill looking, febrile with body temperature of 38.5 degree Celsius, oriented in time, place and person with Glasgow Coma Scale (GCS) of 15/15. Auscultation of the chest revealed decrease breath sounds and bilateral sub‐scapula crackles which were prominent on left side. As shown in Table 1, routine laboratory investigations revealed elevated white blood cell count, elevated erythrocyte sedimentation rate and elevated c‐reactive protein.

**TABLE 1:** Laboratory investigations at presentation.

Complete blood count			Biochemical workup		
Name	Result	Normal range	Test name	Result	Normal range
Hb	10.0 g/dL	12–16 g/dL.	CRP	27.9 mg/dL	<1 mg/dL
M.C.V	58.3 fl	80–10 fl	Serum Creatinine	1.0 mmol/L	5–11 mg/dL
M.C.H	20.1 pg	26–32.5 PG	S. Sodium	140 mmol/L	135–147 mmol/L
M.C.H.C W.B.C	32.1 g/dL 18.4 × 10^9^/L	32–36 g/dL 4–11 10^9^/L	S. Potassium	4.1 mmol/L	3.5–5.5 mEq/L
Neutrophils	89.1%		S. Total bilirubin	0.6 mg/dL	0.1–1.2 mg/dL
Lymphocytes	3.6%		S. Direct bilirubin	0.2 mg/dL	<0.3 mg/dL
Eosinophils	0.5%		S. indirect bilirubin	0.4 mg/dL	<0.2 mg/dL
Monocytes	6.6%		SGPT	84 IU/L	7 to 56 IU/L
Basophils	0.3%				
ESR	70 m.m./hr				

Abbreviations: ESR, erythrocyte sedimentation rate; fl, femtolitre; g/dL, gram per deciliter; Hb, Hemoglobin; IU/liter, international unit/Liter; MCV, mean corpuscular volume; MCH, mean corpuscular hemoglobin; MCHC, mean corpuscular hemoglobin concentration; mm/hr, millimeter/hours; mmol/L, millimole/liter; g/dL, gram per deciliter; mg/dl, milligram per deciliter; pg, picogram; WB, White blood cell count.

**FIGURE 1:**
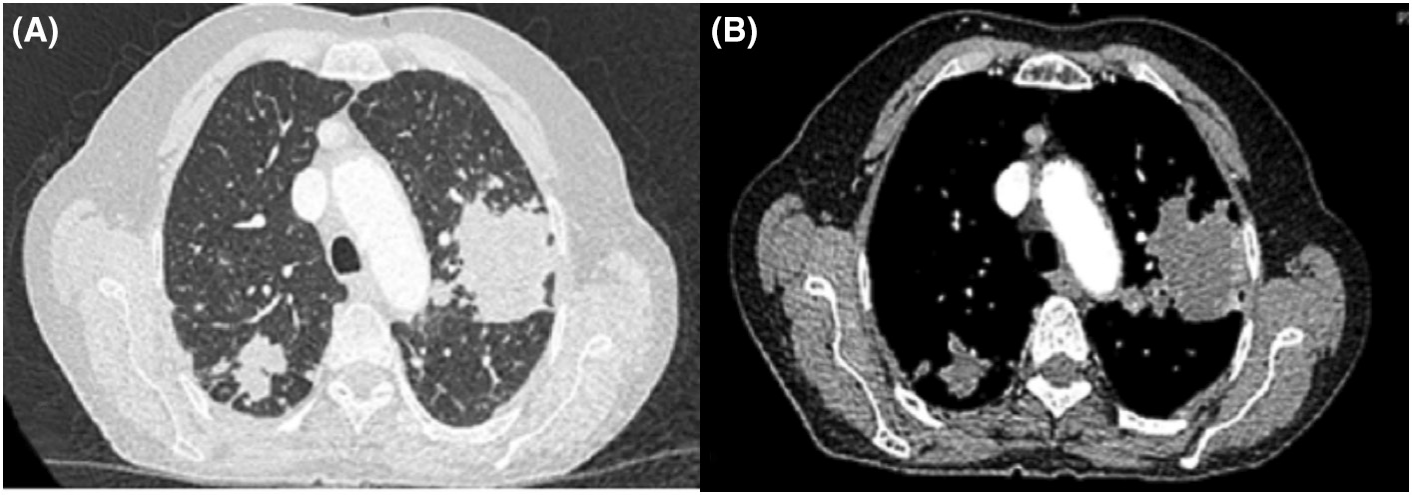
(A) Chest CT, axial cut, lung window: multifocal patchy consolidation with surrounding nodular opacity in both lungs. The largest is in the left lung in the upper lobe which shows relatively speculated outlines. No significantair bronchogram is noted. Centriacinar emphysematous changes of the lungs are seen. (B) Contrast enhanced chest CT, axial cut, mediastinal window: multifocal patchy non‐enhancing consolidation with surrounding nodular opacity. No significant air bronchogram is noted. Irregular speculated outlines of the opacities are seen.

Accordingly, CT chest was advised which was reported to have feature suggestive of left lung upper lobe tumor, multiple hilar and mediastinal lymphadenopathies with bilateral emphysematous changes, as shown in Figure 1. Considering the above‐mentioned findings, the patient was referred to oncologist as well as pulmonologist for re‐evaluation.

### 2.1 Bronchoscopy

**FIGURE 2:**
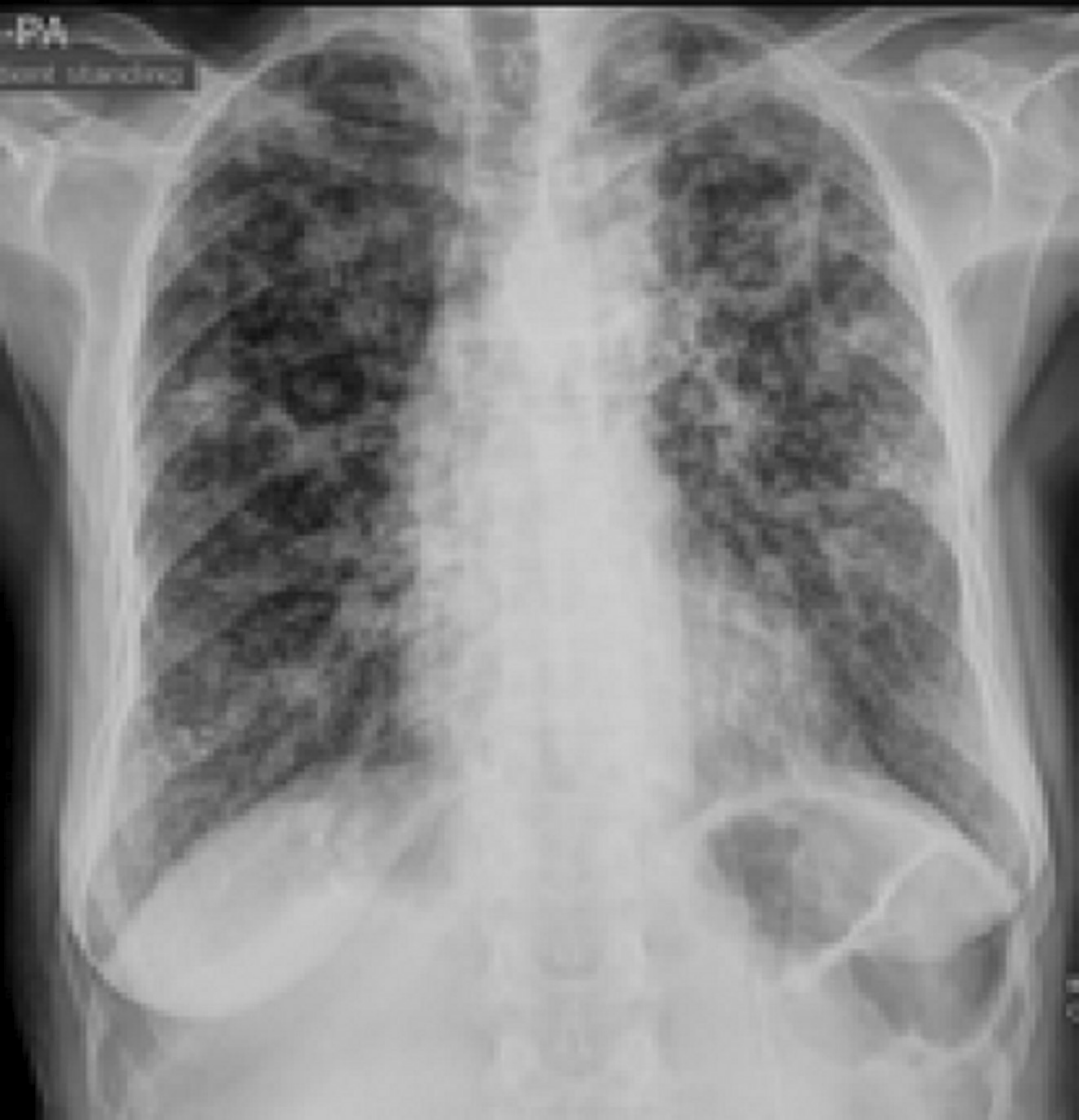
Chest radiograph, frontal projection (post treatment): The previously seen large opacity is resolved. Bilateral emphysematous changes are seen.

Diagnostic bronchoscopy was done for the patient and multiple endobronchial tissue biopsies were taken from the lesion for histopathology. Similarly, Broncho‐alveolar lavage was taken for cytology which was further processed through Gene‐Xpert polymerase chain reaction (PCR) for mycobacterium tuberculosis (MTB), fungal culture, gram stain, and bacterial culture. Biopsy revealed fibro‐collagenous tissue showing predominantly anthracosis, benign respiratory epithelial cells, and focal lung parenchyma showing mild mixed acute on chronic inflammation. No evidence of malignancy or granuloma was seen in the available biopsy. Bronchial wash for cytology revealed RBCs, few neutrophils, rare eosinophils, few macrophages, few ciliated columnar epithelial cells showing reactive atypia, and rare benign squamous epithelial cells without any evidence of granuloma or malignancy. MTB was not detected in the bronchial wash by PCR. There was no evidence of fungal growth in the microbiological cultures. Bacterial culture was positive that demonstrated presence of gram‐negative bacilli and the Analytical Profile Index (API) panel identified the bacteria as *P. mirabilis*. The antibiotic susceptibility is shown in Table 2. The patient was admitted in the inpatient ward and started on parenteral Ertapenem, 1 g once a day with supportive treatment that was continued for 10 days. The patient had significant resolution of symptoms and therefore was discharged from the ward to be allowed to go back home while continuing with oral Ciprofloxacin 500‐mg twice a day for 30 days, bronchodilators and antihypertensive. At the follow‐up visit, the patient reported feeling well with no active complaints. Her vital signs were stable, and her chest X‐ray showed complete resolution of the nonhomogeneous opacity, with only a small pneumatocele visible in the left upper zone. After 1 month, the patient had another follow‐up visit, when she was having no active complaint normal CBC values, normal C‐reactive protein and a repeated chest X‐ray demonstrated total clearing of the opacity and only had residual emphysematous changes, as shown in the Figure 2.

**TABLE 2:** Antibiotic susceptibility testing.

Ertapenem	S	Clindamycin	R
Tazobactam	S	Augmentin	R
Ciprofloxacin	S	Ceftriaxone	R
Levofloxacin	S	Ceftazidime	R
Imipenem	S	Cefipime	R
Meropenem	S	Tobramycin	R
Ampicillin	R	Colistin sulphate	R
Moxifloxacin	R	Cefradine	R
Amikacin	R	Metronidazol	R
Aztreonam	R	SMX/TMP	R

Abbreviation: R, resistant; S, Sensitive; SMX/TMP, sulphamethoxazole/trimethoprim.

## 3 DISCUSSION

In 1885, the swarming nature of microorganism was described by Hauser and based on the speed of their ability to liquefy gelatin, he introduced two species of this strains, that are Proteus vulgaris liquefies gelatin “rapidly,” and *P. mirabilis* which liquefies gelatin gradually.^1^ Among gram negative pathogens, *P. mirabilis* is one of the common pathogen that cause different community or hospital acquired disease, most commonly involving the urinary tract.^6^ The virulence of this bacterium is not very high, however in opportunistically and in immunosuppressed patients *P. mirabilis* can causes wound infection, peritonitis, urinary tract infections, biliary tract infections that can lead to systemic infections.^5^ Very rarely *P. mirabilis* can cause respiratory infection including pneumonia. *P. mirabilis* pneumonia occurs sporadically and does not cause any outbreaks, however 13 cases in a single hospital were reported.^7^ Another study reported 12 cases of multi‐drug resistant extended spectrum beta lactamase (ESBL) producing *P. mirabilis* strains that were isolated from admitted patients in a hospital, of which seven cases were pneumonia.^8^

*P. mirabilis* consists of 3% of all human infection that is a matter of concern, as these cases are multidrug resistant and their treatment and eradication from the body can be very challenging.^9^ *P. mirabilis* is generally resistant to antibiotic such as ceftazidime, cefepime, and fluoroquinolones.^6^ Therefore, for ESBL positive microorganisms carbapenems should be the drug of the choice.^5^ *P. mirabilis* causes pneumonia in patients with chronic debilitation, chronic lung disease, in alcoholics, long term care fascilities, in individuals with renal failure and frail elderly individuals.^9^

One of the complications of acute pneumonia by *P. mirabilis* is formation of pneumatocele in involved lung. Pneomatocele could be transient and spontaneously resolving.^10^ The exact pathogenesis of a pneumatocele is not very clear, however according to one hypothesis, the irritation and inflammation of small bronchioles is thought to trigger formation of a mucus flap, which alternately opens and closes the bronchiolar orifice, effectively acting as a check‐valve.^11^ After resolution of the mucus check‐valve by mucolytics and antibiotics, the stretched alveoli can be recovery.^11^ In some cases such as tension pneumatocele, pneumothorax and infected pneumatocele, invasive interventions may need to be considered.^12^ ESBL‐producing *P. mirabilis* pneumonia that causes pneomatocele is extremely rare and only one case has been reported so far.^10^

On occasion, *P. mirabilis* may cause co‐infection with other organisms, such as Haemophilus influenza. Such co‐infections are especially true in nosocomial settings.^13^ In such cases due to development of biofilm as well as being of nosocomial origin, the presence of multidrug resistance renders the disease to be difficult to treat.^13^ Antibiotic therapy should be dictated by the culture results, such as blood cultures and/ or culture of specimen from other sources, such as bronchoalveolar lavage in our patient. It has been demonstrated that inadequate antibiotic therapy especially when flouroquinolones are used, can result in emergence of multidrug resistant strains of *P. mirabilis*. In such cases antibiotic combination can be utilized to overcome resistant strains.^14^

In our patient, the initial presentation and radiological findings were suggestive of malignant tumor involving the lungs. Re‐evaluation of the case, including broncho‐alveolar lavage and multiple endo‐bronchial biopsies lead to the correct diagnosis of the disease and considering the antibiotic sensitivity profile of the organism, our patient was successfully treated with intravenous Ertapenem for 10 days followed by a long course of ciprofloxacin.

## 4 CONCLUSION

In this report we described a very rare presentation of *P. mirabilis* pneumonia, which clinically and radiologically mimicked malignant pulmonary disease. Multidisciplinary approach is the key towards correct diagnosis, when it comes to the management of lesions suspected for malignancy.

## AUTHOR CONTRIBUTIONS

**Saif Ullah:** Investigation; supervision; validation; visualization; writing – original draft; writing – review and editing. **Ramin Saadaat:** Conceptualization; formal analysis; resources; software; supervision; writing – original draft. **Hidayatullah Hamidi:** Resources; software; supervision; validation; visualization; writing – original draft. **Ahmed Maseh Haidary:** Investigation; resources; supervision; validation; visualization; writing – original draft; writing – review and editing.

## FUNDING INFORMATION

The authors received no funding for current writing.

## CONFLICT OF INTEREST STATEMENT

The authors declare to have no competing interests.

## ETHICS STATEMENT

Ethical approval was acquired from the hospital's ethical review committee. Informed consent was obtained from the patient's legal guardian (father) for participation in the current case report.

## CONSENT

Written informed consent was obtained from the patient's legal guardian for publication of this case report and any accompanying images. A copy of the written consent shall be made available for review by the Editor‐in‐Chief of this journal, upon reasonable request.

## Data Availability

All generated data is included in this article.
